# Abrupt Holocene climate shifts in coastal East Asia, including the 8.2 ka, 4.2 ka, and 2.8 ka BP events, and societal responses on the Korean peninsula

**DOI:** 10.1038/s41598-019-47264-8

**Published:** 2019-07-25

**Authors:** Jungjae Park, Jinheum Park, Sangheon Yi, Jin Cheul Kim, Eunmi Lee, Jieun Choi

**Affiliations:** 1 Department of Geography, Seoul National University, 1 Gwanak-ro, Gwanak-gu, Seoul 08826 Republic of Korea; 2 Institute for Korean Regional Studies, Seoul National University, 1 Gwanak-ro, Gwanak-gu, Seoul 08826 Republic of Korea; 3 Geology Division, Korea Institute of Geoscience and Mineral Resources, 124 Gwahak-ro, Yuseong-gu, Daejon 34132 Republic of Korea; 4 Department of Petroleum Resources Technology, University of Science and Technology, 217 Gajeong-ro, Yuseong-gu, Daejeon 34113 Republic of Korea

**Keywords:** Palaeoecology, Environmental impact, Palaeoclimate, Palaeoecology, Environmental impact

## Abstract

Holocene abrupt cooling events have long attracted attention in academia due to public concern that similar rapid changes may reappear in the near future. Thus, considerable progress has been made toward understanding these short-term cooling events in the Northern Hemisphere, particularly in Europe and North America. However, few relevant studies have been conducted in coastal East Asia due to a lack of undisturbed sample materials appropriate for paleoclimate studies. In this study, we examined Holocene abrupt drying events and the Holocene climate optimum (HCO) based on a new high-resolution multi-proxy record (pollen, mean grain size, total organic carbon, carbon/nitrogen ratio) from the south coast of Korea. Possible cultural impacts of the events were also explored using summed probability distributions (SPDs) of archaeological radiocarbon dates. Our arboreal pollen percentage (AP) data clearly indicated drying events centered at 9.8 ka, 9.2 ka, 8.2 ka, 4.7 ka, 4.2 ka, 3.7 ka, 3.2 ka, 2.8 ka, and 2.4 ka BP. The AP data also indicated that forests were severely damaged by a two-step successive drying event during the period from 8.4 ka to 8 ka BP and that the HCO lasted from ca. 7.6 ka to ca. 4.8 ka BP. According to the results of a correlation analysis, climate variations on the Korean peninsula were possibly controlled by shifts in western tropical Pacific (WTP) sea surface temperatures during the past ~5500 years. Simultaneous declines in the SPDs and AP from 2.8 ka to 2.3 ka BP may reflect a demographic reduction attributable to rapid climate deterioration on the peninsula. Refugee agriculturalists might have immigrated to Japan and developed the Yayoi culture. In this study, the 2.8 ka event and its societal impact are recognized clearly for the first time in coastal East Asia.

## Introduction

Holocene abrupt cooling events have been vigorously investigated due to concerns that similarly rapid shifts may appear again in the future^1,2^. Early Holocene cooling events such as the 8.2 ka event are believed to have been initiated by meltwater drainage from proglacial lakes into the North Atlantic^3–5^. If global warming accelerates more rapidly, increased melting of the Greenland ice sheet might give rise to another cooling event in the Northern Hemisphere (NH)^6–8^. Coastal East Asia, including eastern China, Korea, and Japan, is one of the world’s most populous regions, and is thus vulnerable to abrupt environmental change. Therefore, an accurate understanding of past climate variations is particularly important in this region.

Holocene climate research from East Asia has mainly focused on the following topics: 1) changes in the East Asian monsoon (EAM) and the mechanisms involved^9,10^; 2) the Holocene climate optimum (HCO)^11,12^; 3) abrupt Holocene climate events such as the 8.2 ka and 4.2 ka BP events and Little Ice Age (LIA)^13,14^; and 4) a link between climate deteriorations and decline in ancient societies^15,16^. The dynamics that underpin the spatial and temporal differences in the Holocene EAM variability are still not clearly understood^11,12,17–19^, and further investigation is needed. There are currently concerns that Chinese cave δ^18^O records, long considered the most reliable proxy of Holocene EAM, have not been properly interpreted^20–22^. However, based on various paleoclimate proxy data and modelling results, it is generally agreed that variations in orbital insolation are mainly responsible for the long-term evolution of Holocene EAM intensity^23^.

However, the detailed mechanistic explanation of short-term Holocene cooling events is apparently incomplete in East Asia as the underlying causes behind them are too complicated to be clearly identified. Abrupt Holocene cooling events in the NH are believed to have been caused by many different factors, for example, variations in the Atlantic meridional overturning circulation (AMOC), tropical Pacific sea surface temperature (SST), volcanic activity and sunspot activity. Furthermore, the principal causes of these climate shifts seem to have varied over time during the Holocene^24^.

Cave stalagmite δ^18^O records from inland China are detailed enough to investigate the timing and structure of abrupt Holocene climate events, and δ^18^O data with sound chronologies and high temporal resolutions provide reliable and useful paleoclimate information. However, recent studies suggest that there was a considerable difference in late Holocene climate change between inland China and coastal East Asia^25,26^. Thus, as many local records as possible are needed to better understand the causes of abrupt climate shifts and to assess the societal response more accurately.

The influence of the 4.2 ka event on past societies is currently one of the most intriguing topics among paleoclimatologists and archaeologists in coastal East Asia^16,27–33^. Also, the LIA has been extensively discussed and investigated in academia, to the point that the public also knows it well^34^. However, few studies have been performed on early Holocene cooling events, and convincing evidence of the 8.2 ka event was not reported in coastal East Asia until very recently^35^.

Other late Holocene abrupt cooling events in addition to the 4.2 ka and LIA are also deserving of careful attention but have rarely been explored in this region. Paleoclimatological and archaeological evidence from Eurasia indicates that the natural environment and human societies were substantially influenced by an abrupt climate change around 2.8 ka BP^36–38^. This cooling event is suggested to have arisen from a solar-induced shift in atmospheric circulation^39^. Some scholars have argued that a 3.2 ka megadrought led to the collapse of ancient societies in the eastern Mediterranean^40–42^. However, there has been little investigation of the influences that these cooling events exerted on the ancient societies of coastal East Asia.

Holocene paleoclimate studies from China demonstrate that the timing and duration of the HCO significantly varied in East Asia^11,12,43^. Furthermore, the transition was not sudden but rather gradual, so the onset and termination are not clear. Therefore, there has long been a debate regarding the spatial and temporal dynamics of the HCO in East Asia^12,18^. However, in Korea, the HCO has never been properly discussed due to a lack of well-dated local proxy data spanning the entire Holocene.

In this study, we present a new high-resolution multi-proxy record (pollen, total organic carbon (TOC), total organic carbon/total nitrogen (C/N) ratio, mean grain size) of the Holocene based on a ~30-m long sediment core from an estuarine floodplain of Seomjin River, South Korea. The aims of the study were to (1) reconstruct the Holocene climate and vegetation history in the study area, (2) examine the abrupt drying events and the HCO, and (3) explore the possible impacts of the events on ancient human societies.

## Study Area

**Figure 1:**
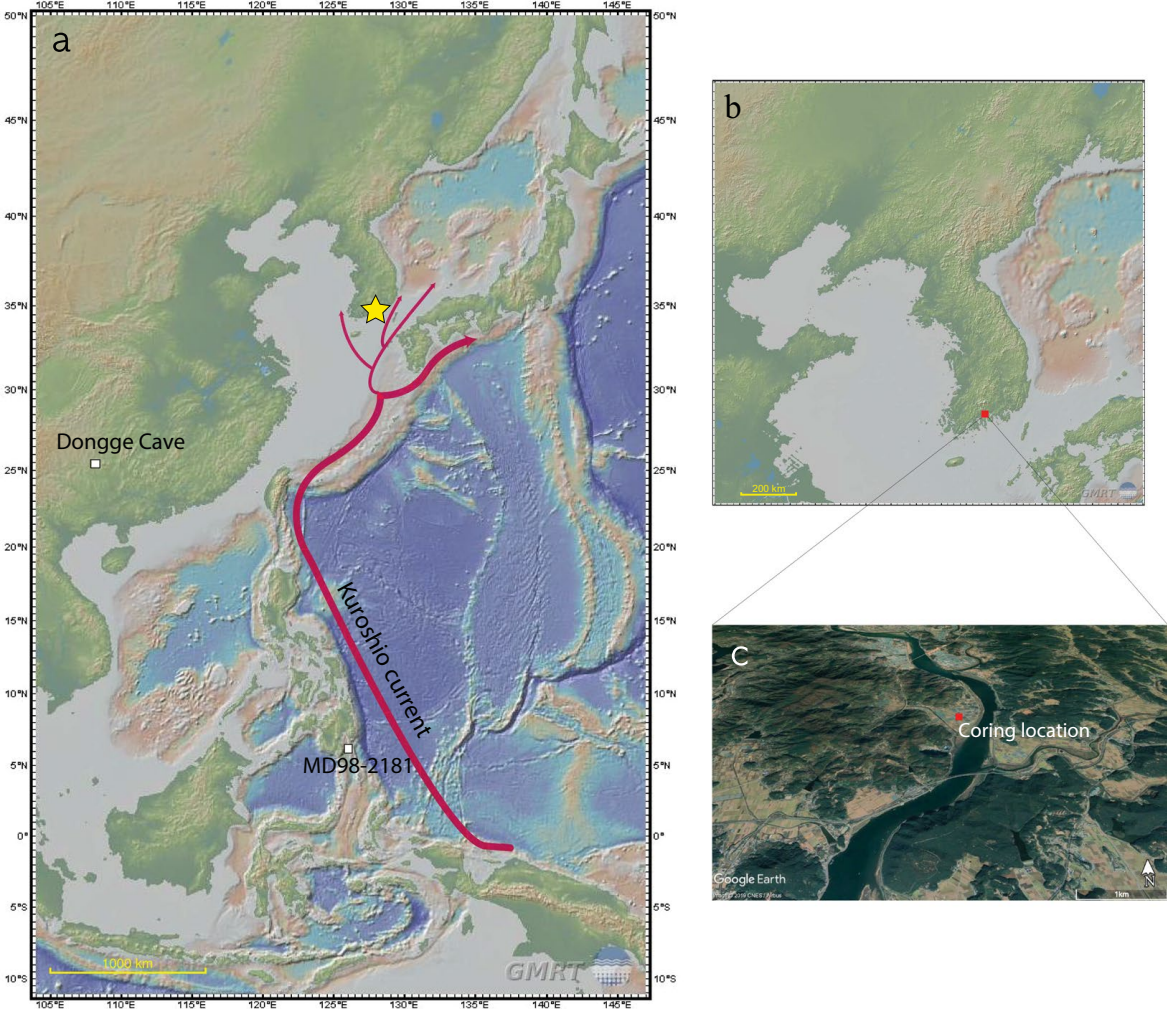
(**a**) Locations of the study site (yellow star) and paleoclimate records used in this study: site MD98-2181, Mindanao, Philippines^58^ and Dongge Cave, southern China^57^ (See Fig. 7). (**b**) Location of the coring site (red square), Gwangyang-si, South Korea. These two location maps were created using GMRT Map tool (www.gmrt.org/GMRTMapTool/)^115^. (**c**) Coring location (red square). The bird’s-eye view image was generated using Google Earth (www.google.co.kr/intl/ko/earth/).

Our study site, an estuarine floodplain of Seomjin River, is located in Jinwol-myeon, Gwangyang-si, Jeollanam-do, on the central south coast of the Korean peninsula (Fig. 1). The Seomjin River is the second largest river without a tidal embankment in Korea, and its ecological conservation is therefore essential^44^. This river is characterized by its flows in deep valleys between relatively steep hills, unlike other large rivers in Korea. Therefore, large plains are not seen in the estuary. The narrow estuarine floodplains have mostly been reclaimed and converted into rice paddies.

Korea has four distinct seasons, with a large difference in monthly average temperatures between summer and winter. Precipitation is predominantly concentrated in summer. The peninsula lies in the EAM belt, and the EAM is the dominant factor influencing the climate in this region. The summer (winter) monsoon brings hot (cold) and humid (dry) weather to the Korean peninsula. Larger seasonal contrasts in atmospheric temperature over the continent and smaller differences over the ocean result in seasonal shifts of prevailing winds from southeast in summer to northwest in winter. The study site, located in one of the rainiest places in the country, is relatively humid in summer and warm in winter compared to other inland areas. The coldest mean monthly temperature at the Yeosu station, ~25 km from the study site, was 2.4 °C in January, while the warmest mean monthly temperature was 25.8 °C in August. The average precipitation in the area is approximately 1440 mm. Summer season rainfall (June–September) averages ~930 mm, accounting for approximately 65% of the annual mean precipitation in the study area^45^.

The potential vegetation in the study area is categorized as warm temperate forest and consists of broadleaved evergreen trees such as *Cyclobalanopsis acuta*, *Castanopsis cuspidata* var. *sieboldii*, *Camelia japonica*, etc.^46^. However, a long history of human disturbance has caused *Pinus densiflora* to become the dominant tree species in the study area. Other common trees include *Pinus thunbergii*, *Populus deltoides*, *Salix chaenomeloides*, *Castanea crenata*, *Aphananthe aspera*, *Celtis sinensis*, and *Maclura tricuspidata*. *Typha angustifolia*, *Paspalum distichum*, *Zizania latifolia*, *Scirpus triqueter*, *Monochoria vaginalis*, and *Trapa natans* are important aquatic plants. *Phragmites communis* is the most dominant salt marsh plant in the study area. *Blitum virgatum*, *Suaeda glauca*, *Suaeda japonica*, *Limonium tetragonum*, *Tripolium pannonicum*, and *Carex scabrifolia* are also common on uncultivated tidal wetland^44^.

## Results and Discussion

### Chronology, stratigraphy, and sedimentary environment

**Figure 2:**
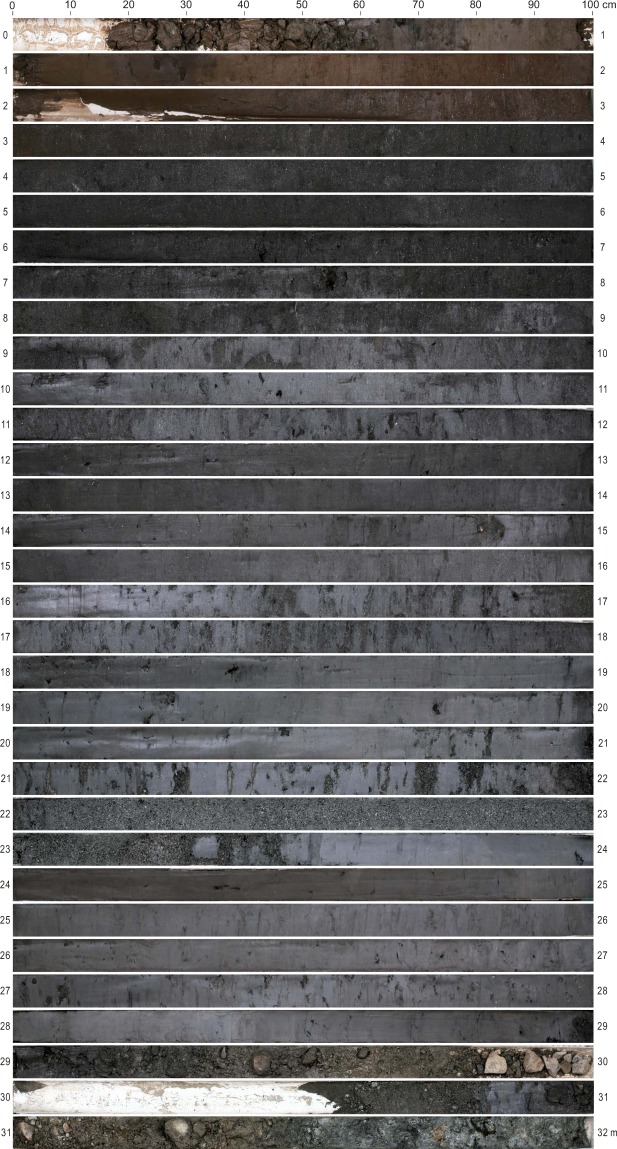
A digital image of the 32-m-long GY-1 sediment core.

**Figure 3:**
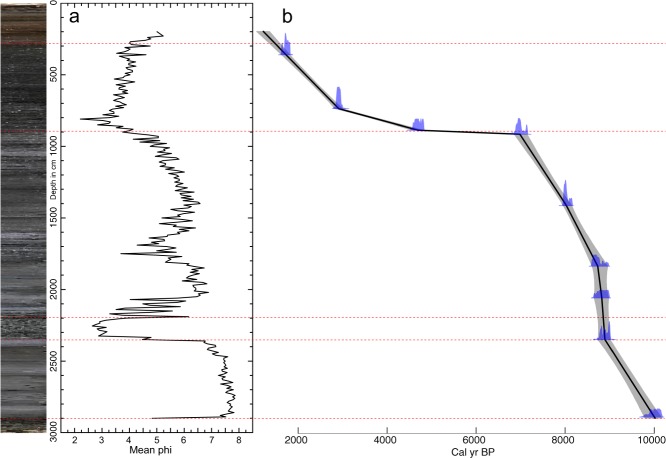
(**a**) Stratigraphy and mean grain size of the GY-1 sediment core. (**b**) GY-1 core age depth profile. The best age model (black line) with a 95% confidence interval (gray shading) was established based on linear interpolation between dated levels using Clam 2.2. Red dotted lines show boundaries of sections divided according to physical properties of sediments and sedimentation rates. This diagram was produced using pro Fit 7.0.7 software (www.quansoft.com).

**Figure 4:**
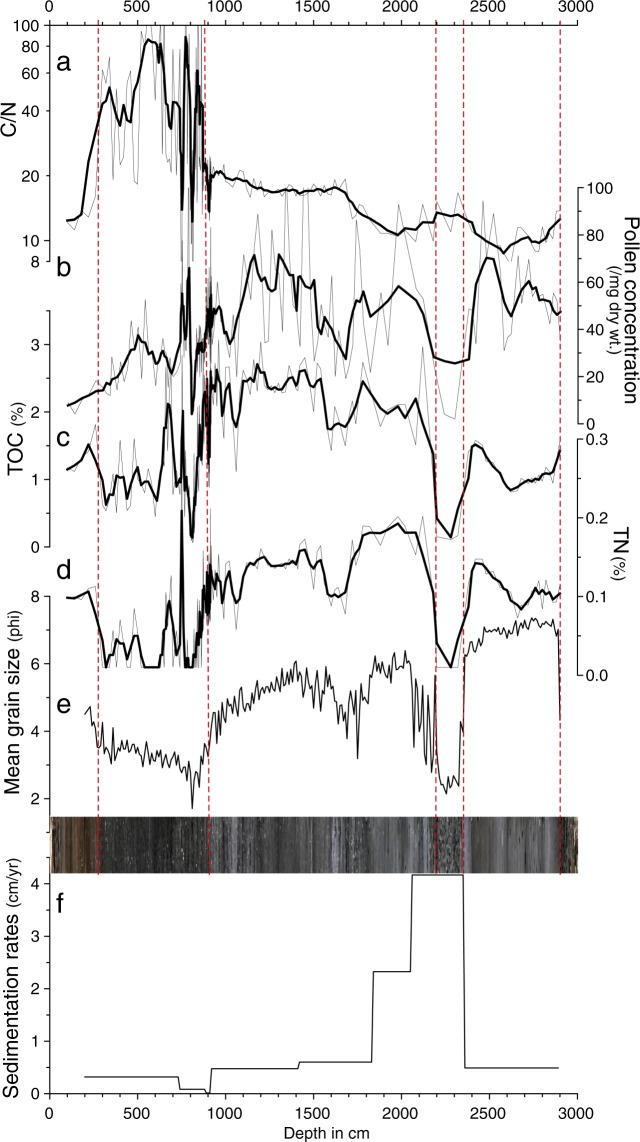
GY-1 sediment total organic carbon/total nitrogen (C/N) ratio (**a**), pollen concentration (**b**), total organic carbon (TOC) (**c**), total nitrogen (**d**), mean grain size (**e**), and stratigraphy and sedimentation rates (**f**). This diagram was produced using pro Fit 7.0.7 software (www.quansoft.com).

In total, nine radiocarbon dates were obtained from the GY-1 sediment core (Table 1; Figs 2 and 3). Large changes in sedimentation rates were observed in the age–depth profile, but there was no age reversal. For the description of sediment stratigraphy, the GY-1 sediment profile was divided into six sections based on the physical properties of sediments and sedimentation rates (Figs 3 and 4). The gravel layer below a depth of 29 m indicates that drilling reached the bedrock (Fig. 2).

**Table 1:** Radiocarbon dates for GY-1 sediments. Calibration was carried out with Clam 2.2 software^110^.

Sample Depth (cm)	Material dated	Laboratory No.	δ^13^C	Age (^14^C yr BP)	Two σ age range (cal yr BP)
363	Plant fragments	KGM-IWd170355	−26.2	1800 +/− 30	1625–1819
739	Plant fragments	KGM-IWd170356	−24.7	2810 +/− 30	2846–2997
889	Plant fragments	KGM-IWd170357	−30.9	4150 +/− 30	4578–4825
917	Plant fragments	KGM-IWd170358	−26.8	6100 +/− 40	6863–7157
1416	Plant fragments	KGM-IWd170359	−26	7210 +/− 40	7956–8158
1839	Plant fragments	KGM-IWd170360	−32.1	7900 +/− 40	8595–8976
2060	Plant fragments	KGM-IWd170361	−27.9	7960 +/− 40	8649–8990
2351	Plant fragments	KGM-IWd170362	−27.4	8030 +/− 40	8764–9024
2892	Plant fragments	KGM-IWd170363	−27.2	8870 +/− 40	9785–10173

Sediments between a depth of 29 m and 23.5 m consist mainly of fine clayey silts. The surface of the sediments generally appears gray in color. Unlike upper sections, shells are hardly visible, while plant fragments are frequently observed. They appear to be almost purely terrestrial (i.e., not estuarine) sediments deposited in a backswamp before being influenced by a sea level rise from 10 ka to 9 ka BP. The relatively high pollen concentration in this section is probably attributable to a slow deposition of clastic materials in the backswamp. Low C/N ratios (~10) indicate that autochthonous freshwater algae were the main sources of sedimentary organic matter. Marine and freshwater algae both have C/N ratios < 10, while terrestrial plants tend to have C/N ratios > 20^47–49^.

The sediments between 23.5 m and 22 m are composed mostly of sand. Sedimentation rates are extremely high, indicating fast accumulation in a short period of time. This sand section may have formed ca. 9 ka BP when the study area was abruptly influenced by rising seawater levels. However, the early Holocene marine transgression seems to have begun relatively late in the study area compared to Biguem Island, offshore southwest Korea^35^. This is probably because our study site is located relatively deep inland from the coast. This sea level rise, often referred to as melt water pulse 1c (MWP-1c), is suggested to have been triggered by deglacial meltwater drainage from Lake Agassiz^50,51^.

The sediments between 22 m and 9 m are mainly composed of silt and their surfaces mostly show darkish gray colors. This section includes sediments deposited under the influence of rapid sea level rise from approximately 9 ka to 7 ka BP. Shell fragments are often seen throughout the section, reflecting a shift from riverine environments to estuarine environments in the study area. High TOC and TN values are also indicative of increasing oceanic influence with higher sea level. The farther from the estuary toward the outer continental shelf, the higher TOC and TN values were, according to a study on sediments recovered off the estuary of Seomjin River^52^. In addition, there is an interesting decrease in mean phi values, TOC, TN, and pollen concentration between 18 m and 16 m. A deposition of such coarser materials, like in the lower section between 23.5 m and 22 m, may have been caused by a sudden elevation in sea level. The subsequent expansion of accommodation space in the sedimentary basin presumably resulted in a decline of sedimentation rates (Fig. 4f). This sea level rise seems to have been associated with another massive Lake Agassiz discharge, which was suggested to have consisted of two episodes^53^. Numerical model studies indicate that the earlier freshwater release at ca. 8.4 ka BP possibly led to a 1-m sea level rise along the coast of East Asia^54^. The later outburst is believed to have been the major cause of the 8.2 ka cooling event^4,51,53^.

The sediments from a depth of 9 m to 3 m are composed of dark grayish fine sand and silt. There is a plateau phase of sedimentation at a depth of 9 m. This indicates that the sea level rise terminated at ca. 7 ka BP, leading to almost no sedimentation until 5 ka BP. High mean phi values around a depth of 8 m are probably indicative of coarse sediments deposited intermittently by rare large-scale floods. Then, as reflected by an upward gradual decrease in sediment particle size (i.e., an increase in mean phi values), our coring site may have become farther away from the river channel. Increased transport of clastic materials from upstream may have caused the floodplain to expand. This is presumably related to upland deforestation arising from climate deterioration during the late Holocene. A related point is that the major sources of sedimentary organic matter abruptly changed from estuarine pools to terrigenous pools, as indicated by a marked increase in C/N ratios from a depth of ~9 m. Very high C/N values are clearly indicative of a shift to terrestrial vascular dominance in organic matter. Above a depth of 3 m, there are brownish sediments disturbed by agricultural activities.

### Pollen records

**Figure 5:**
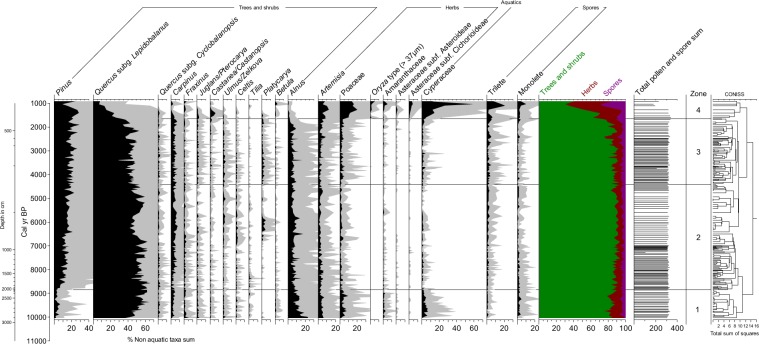
Selected pollen taxa from GY-1 sediments. All percentages are based on the total nonaquatic taxa sum. Gray shading indicates a 5 × exaggeration.

GY-1 pollen assemblages consistently show high percentages of arboreal taxa such as *Pinus*, *Alnus*, and *Quercus*. The latter is particularly important throughout the entire period of investigation. For convenience, the pollen diagrams were divided into four zones based on the clustering results (Fig. 5).

#### Pollen zone 1 (10 ka to 8.9 ka BP)

This zone is characterized by the predominance of *Quercus. Alnus* maintains high percentages, averaging over 10% but steadily decreasing upward. There is also a high relative abundance of wetland plants such as Cyperaceae, and herbs such as *Artemisia*, Poaceae, and Amaranthaceae. By contrast, *Pinus* percentages are consistently very low (<7%). A gradual upward increase in *Quercus* and opposing trends of *Alnus*, *Betula*, *Artemisia*, and Cyperaceae all indicate that the climate became warmer and wetter.

In this zone, *Quercus*:*Pinus* ratios are unexpectedly high, given the relatively high percentages of herbaceous taxa. The predominance of *Quercus* over *Pinus* is usually interpreted as a climate that was relatively warm and wet. However, the high abundances of herbs requires *Quercus*:*Pinus* ratios in this zone to be interpreted differently. As mentioned previously, the study site seems to have been a riverine backswamp during the period corresponding to zone 1 and an estuarine wetland during later periods. Proportions of pine pollen usually increase with distance offshore, since bisaccate pollen types have a better chance to enter the ocean through aerial transport, in addition to fluvial transport^55^. Low pine percentages reflect sparse deposition of marine materials. Zone 1 is mostly dominated by pollen dispersed over relatively short distances from local plants.

#### Pollen zone 2 (8.9 ka to 4.4 ka BP)

Pollen assemblages in zone 2 demonstrate variations in vegetation when rising sea levels exerted profound influences on the study area. *Pinus* percentages increase steeply at the boundary of zone 1 and 2, while *Quercus* percentages decrease. This seems primarily due to a shift in the sedimentary environment, as mentioned above. The abrupt shift at the zonal boundary indicates that the environment changed from a backswamp with steady and slow deposition to an estuarine wetland with rapid sedimentation.

Throughout the zone, *Quercus* dominates and maintains its high percentages (~50%) with no significant change. *Carpinus* slightly increases from the previous zone, while *Alnus* and herbaceous plants all decline. Such shifts in vegetation reflect an ameliorating climate as the HCO began. In addition, a rapid decline in Cyperaceae percentages indicates that the abrupt seawater intrusion led to a decrease in the area of inhabitable wetlands.

#### Pollen zone 3 (4.4 ka to 1.6 ka BP)

In this zone, there is a slight but noticeable decline in *Quercus* from zone 2, and increases in *Pinus*, *Artemisia* and Poaceae. This change indicates that the HCO ended, and the climate deteriorated. An increase in *Platycarya* also demonstrates that under unfavorable climate conditions, climax trees were replaced by shade-intolerant trees.

#### Pollen zone 4 (1.6 ka to 0.9 ka BP)

The proportions of all arboreal taxa, in particular *Quercus*, are greatly reduced in this zone. In contrast, there is a remarkable increase in herbaceous plants such as *Artemisia*, Poaceae, *Oryza* type, Amaranthaceae, and Asteraceae. These changes indicate that the agricultural disturbance began ca. 1.6 ka BP in the study area. Cyperaceae also increases sharply at the beginning of the zone, and it increases even more markedly in the latter part of the zone. This increasing pattern is similarly shown by trilete and monolete spores. This may reflect that the local people, probably due to floods, abandoned floodplain farmlands, which were later inhabited by large populations of sedges and ferns.

### El Niño–Southern Oscillation (ENSO) forcing of Holocene climate and its periodicities

**Figure 6:**
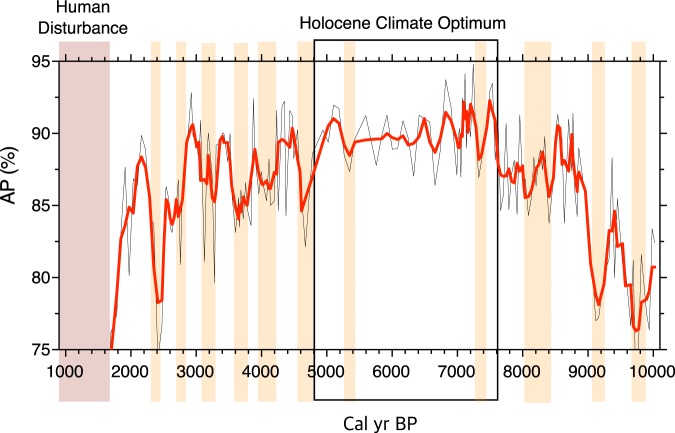
The arboreal pollen percentage (AP) diagram. Drying events are indicated by yellowish boxes; the period of human disturbance is indicated by a reddish-brown box.

**Figure 7:**
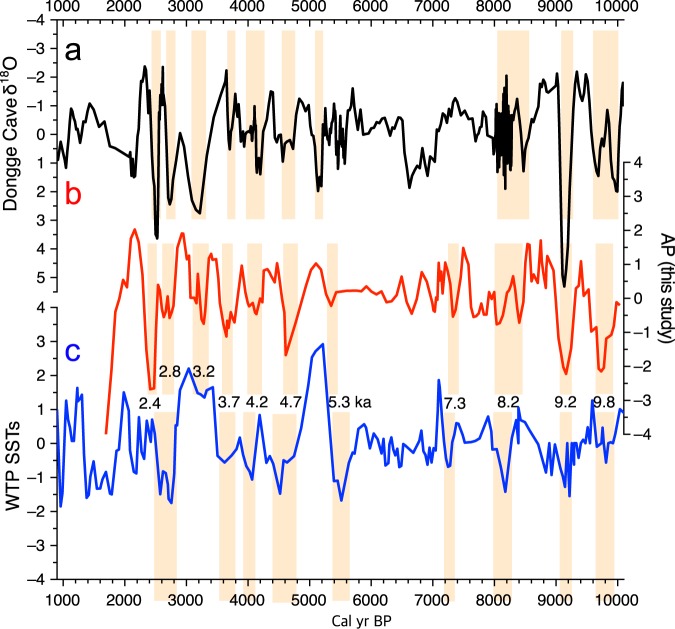
Comparison of the Dongge Cave δ^18^O records^57^ (**a**), arboreal pollen percentages from this study (**b**), and reconstructed sea surface temperatures (SSTs) of the western tropical Pacific (WTP)^58^ (**c**). Drying events are indicated by yellowish boxes, with their approximate ages. All the proxy data were detrended, smoothed, and normalized (see the methods section for details). This diagram was produced using pro Fit 7.0.7 software (www.quansoft.com).

In this study, the pollen record (i.e., pollen percentage (AP)) was only used for the reconstruction of climate change (Fig. 6) because other proxy records mostly indicate changes in the sedimentary environment. Paleoclimate information has so far been successfully extracted from AP variations reported in pollen studies of Holocene EAM^33,35,56^. Also, to better understand the mechanism underlying Holocene climate change in the study area, interregional similarities and differences were examined by comparing normalized paleoclimate proxy data from Dongge Cave, southern China^57^ and the western tropical Pacific (WTP) off the coast of Mindanao, Philippines^58^, with normalized GY-1 AP records (Fig. 7; Table 2). Correlation analyses showed a weak correlation between the AP data and Dongge δ^18^O data (r = −0.20) and a stronger correlation between the AP data and the WTP SST reconstruction (r = 0.29).

**Table 2:** The correlations between our AP data and Dongge δ^18^O data^57^ and WTP SST reconstruction^58^. These proxy records were all detrended, smoothed, and normalized. The numbers in parenthesis represent the 95% confidence interval calculated by Pearson T3, designed for estimating the correlation between unevenly-spaced auto-correlated data^114^

Time period		GY-1 AP
1.6 ka–10.2 ka	Dongge δ^18^O	−0.204 (−0.582; 0.247)
	WTP SSTs	0.287 (0.142; 0.419)
1.6 ka–5.6 ka	Dongge δ^18^O	0.053 (−0.149; 0.251)
	WTP SSTs	0.346 (0.040; 0.592)
5.6 ka–10.2 ka	Dongge δ^18^O	−0.543(−0.846; −0.024)
	WTP SSTs	0.156 (−0.013; 0.317)

In addition, all proxy records were divided into two datasets (before 5.6 ka and after 5.6 ka BP) to assess the change in the strength of correlations attributable to enhanced ENSO forcing after ca. 5.5 ka BP (Table 2). The latter is generally believed to have been caused by a decline in NH insolation during the late Holocene^59–61^, although some recent records are indicative of reduced ENSO intensity between 5 and 3 ka BP, which was not in phase with the insolation change^62,63^. Before 5.6 ka BP, there was a relatively strong correlation between the AP data and the Dongge δ^18^O data (r = −0.54, 95% confidence interval (–0.85; –0.02)) and a much weaker correlation between the AP data and the WTP SST reconstruction (r = 0.16, 95% confidence interval (–0.01; 0.32)). However, after 5.6 ka BP, a significant increase in the correlation was found between the AP data and the WTP SST (r = 0.35, 95% confidence interval (0.04; 0.60)), coupled with a substantial decline in the correlation between the AP data and the Dongge δ^18^O data (r = 0.05, 95% confidence interval (–0.15; 0.25)). These results may indicate that climate change in the study area was mainly modulated by tropical ocean forcing during the last 5000–6000 years.

**Figure 8:**
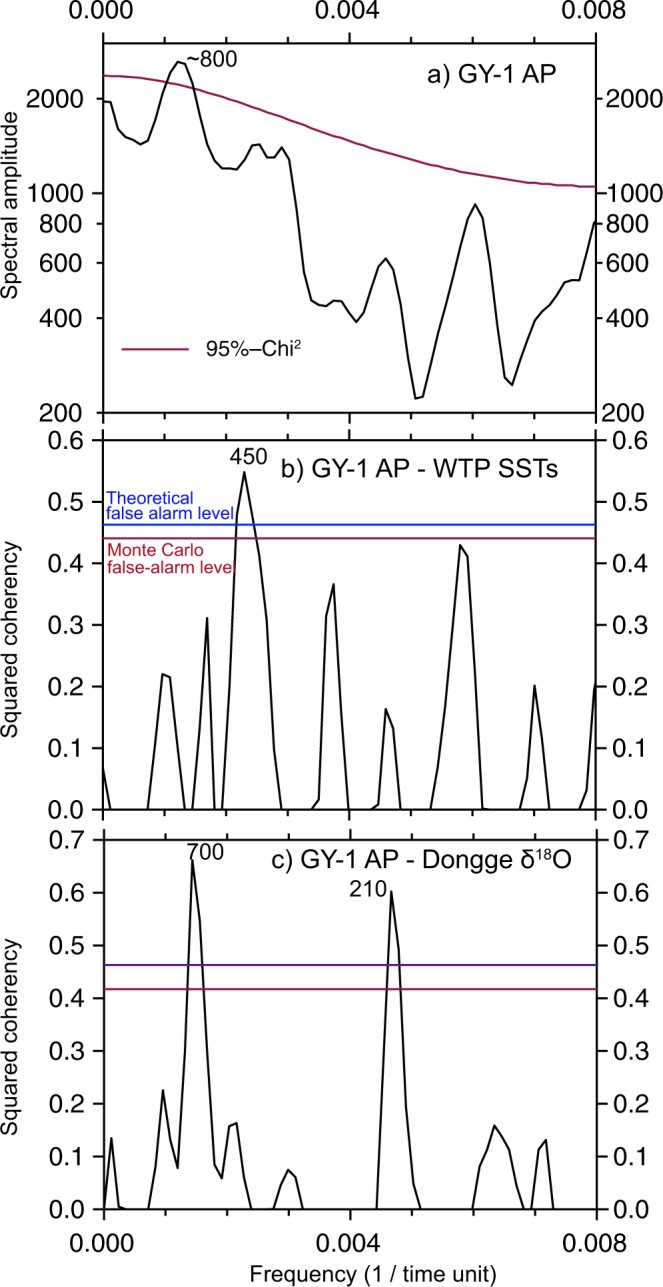
Auto spectral analysis of the GY-1 arboreal pollen percentage (AP) (**a**). Cross spectral analyses between GY-1 AP and western tropical Pacific sea surface temperature (WTP SST) reconstruction data (**b**) and between GY-1 AP and Dongge δ^18^O data (**c**). Significant periodicities are labelled in years. This diagram was produced using pro Fit 7.0.7 software (www.quansoft.com).

An autospectral analysis revealed a significant periodicity of ~800 years, with 95% confidence, in our AP data (Fig. 8a). Similar periodicities have been frequently reported around the study area^64,65^. The periodicities seem to be in harmony with the ~1500-year cycle in the NH, which is believed to be mainly attributable to Holocene AMOC shifts^66–68^. According to cross-spectral analysis results (Fig. 8b), the periodicity of 450 years was significant at the mean Monte Carlo false alarm level (α = 0.05) between the AP data and the WTP SST reconstruction. The 450-year cycle was related to the 500-year cycle verified in the reconstruction of solar activity^69^. Its existence in East Asia was recently identified by terrestrial sediment proxy records^65,70,71^ and oceanic records^64,72^. Between the AP data and Dongge δ^18^O data, periodicities of 700 and 210 years were verified in the coherency spectrum (Fig. 8c). The periodicity of 210 years is a well-established solar cycle inferred from records of cosmogenic nuclides^69^ and is one of the most often encountered patterns in East Asia^73^.

### Holocene climate optimum and abrupt drying events

As mentioned earlier, the HCO of the Korean peninsula could not be properly investigated because of the lack of well-dated, high-resolution proxy records^74^. Its timing and nature have thus remained unclarified for decades. However, for the first time in Korea, our AP records show a clear signal of the HCO (7.6 ka to 4.8 ka BP) (Fig. 6). Although the results of a cluster analysis based on the pollen composition indicated that optimum climatic conditions may have lasted from 8.9 ka to 4.4 ka BP, its core period is likely to have existed between 7.6 ka and 4.8 ka BP. This is not much different from the HCO interval (ca. 8 ka to ca. 5 ka BP) shown in Chinese pollen records at similar latitudes^12^.

Our results also provide information on abrupt short-term events that quasi-periodically appeared during the Holocene. The AP data demonstrated almost all of the abrupt events widely known throughout the NH. During the early Holocene, these short-term drying events likely recurred approximately every 1000 years in the study area. Many NH paleoclimate studies have reported similar shifts in early Holocene climate, for example, events centered at 9.8 ka^75,76^, 9.2 ka^77,78^, 8.2 ka^13,79^, 7.3 ka^80^, and 5.3 ka BP^81,82^.

However, late Holocene climate change from 5 ka to 2 ka BP was mainly modulated by a roughly 500-year cycle, leading to abrupt climate deteriorations at 4.7 ka, 4.2 ka, 3.7 ka, 3.2 ka, 2.8 ka, and 2.4 ka BP in the study area. These events have also been long identified in various NH proxy records, for example, 4.7 ka^83^, 4.2 ka^84–86^, 3.7 ka^87^, 3.2 ka^40^, 2.8 ka^88–90^, and 2.4 ka BP^91,92^.

Most researchers agree that early Holocene cold events were triggered by proglacial megaflooding at the margin of the Laurentide ice sheet. However, there is no general agreement on the causes of cooling events occurring after the complete disappearance of the ice sheet^24,83^. Late Holocene cooling events may have been driven by increases in North Atlantic Arctic drift ice, induced by a quasiperiodic decline in sunspot activity. Diluted sea waters would have slowed the AMOC, leading to the cooling of the NH^66^.

As shown in Fig. 7, the short-term shifts in the three proxy datasets correspond well with each other, although there are some discrepancies. For example rapid climate amelioration occurred immediately after the 8.2 ka event in East Asia, as indicated in Dongge Cave δ^18^O records; however, Korean forests remained damaged and in disequilibrium with the prevailing climate for as much as 400 years. A tremendous impact was probably exerted on the Korean forests by a short-term double-drying event during 8.4 ka to 8 ka BP, which is clearly identified in other regions as well^9,13,57,93^. The decline of arboreal taxa during drying events implied that tree flowering, and thus pollen production, were depressed by rapid climate deterioration and/or elevated vulnerability to infectious disease^94,95^. However, climate deterioration around 8.2 ka BP was especially prominent, and many trees seem to have failed to survive it, not to mention that pollen production was severely reduced. The sizable impacts of the 8.2 ka event and its outcomes are apparently reflected by a 400-year delay in recovery of forest ecosystems.

Abrupt climate shifts, which were vaguely seen during the HCO, reappear from ca. 5 ka BP in all three proxy datasets. The tendency and variation shown in late Holocene AP records were more similar to those in the WTP SST reconstruction^58^ than Dongge δ^18^O records^57^. In particular, the 4.7 ka event, indicating the termination of the HCO in the Korean peninsula, is more clearly demonstrated in reconstructed WTP SSTs. The temporal coincidence between low AP values and decreased WTP SSTs indicates that a decline in precipitation adversely affected tree growth. Decreasing SSTs are likely to have hampered the formation of strong cyclones over the western North Pacific, and thus reduced moisture transfer to the Korean peninsula^26^.

### Impact of Holocene abrupt climate events on ancient societies

**Figure 9:**
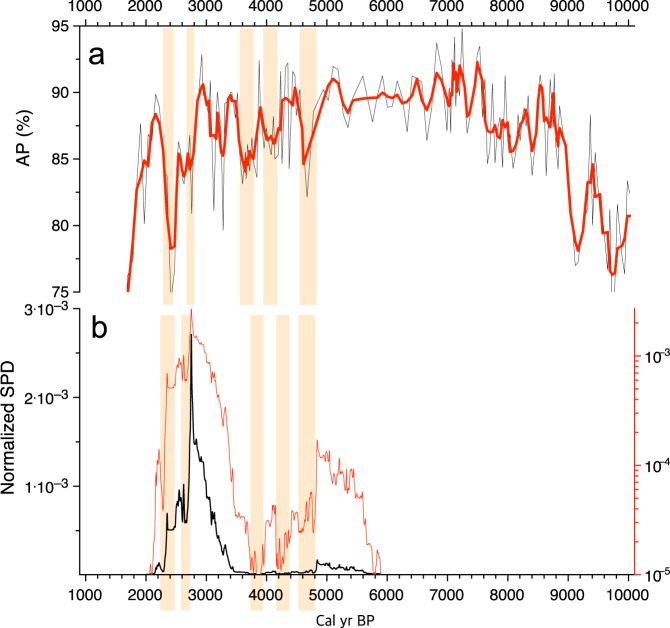
Comparison of the GY-1 arboreal pollen percentage (AP) (**a**) and normalized summed probability distribution (SPD) of radiocarbon dates^98^ (**b**). Drying events are indicated by yellowish boxes. The red curve in (**b**) represents logarithmic SPD values. Note the log scale on the right Y-axis. This diagram was produced using pro Fit 7.0.7 software (www.quansoft.com).

In recent archaeological studies, summed probability distributions (SPDs) of ^14^C dates have been increasingly used as proxy data for the changes in population size of ancient societies^96–99^. We compared our AP data with Korean SPD data spanning the period from 5.9 ka to 2.2 ka BP^98^ to examine the impact of late Holocene abrupt events on ancient societies (Fig. 9). The SPD data were produced using 2190 dates of plant materials excavated from pit houses in 513 settlements. Charcoals accounted for ~94% of the total sample. Other detailed information about the SPD data is provided by Oh *et al*.^98^.

The AP values rapidly decrease ca. 4.7 ka BP at the termination of the HCO; at which time the SPD values also decrease abruptly. Then, the AP values show two successive steps in decline from ca. 4.2 ka to ca. 3.6 ka BP. The SPD values also show a similar declining trend, but with slight temporal differences. This seems to be a matter of dating; however, they might not have been related to each other. Careful attention is needed so as to not give too much meaning to small variations, because there are unavoidable flaws in the SPD data, possibly arising from sample biases, and the variety of research objectives and calibration processes^100,101^. Thus, only broad trends in population change may be determined from the SPD data^98^. However, given the general similarity in declining trends, it seems plausible to suggest that climate deterioration was associated with a change in population between 4.7 ka and 3.5 ka BP. Abrupt drying and/or cooling events probably reduced the availability of resources such as acorns, as reflected by decline in *Quercus* pollen percentages. Residential mobility would have inevitably been escalated, acerbated by a shortage of food, resulting in the construction of fewer dwelling structures. This scenario of elevated mobility is well supported by archeological findings that outdoor features substantially increased in number between ca. 5 ka BP and ca. 3.5 ka BP^102^.

A dramatic rise in the SPD from ca. 3.5 ka BP reflects a soaring population as rice agriculture began on the peninsula. Ameliorating climate conditions, indicated by the simultaneous increase in AP values, would have been beneficial for new farmers to grow a subtropical rice crop. However, SPD values dropped drastically at ca. 2.8 ka BP, and AP values also showed a marked drop. An agrarian subsistence economy may have been heavily undermined by an abrupt drying and/or cooling event. Worsening climate conditions were presumably a great threat to a society of early agriculturalists given its growing population and poor agricultural technologies. Most rice farmers seem to have finally abandoned their sedentary lifestyle around 2.4 ka BP when they faced another abrupt drying and/or cooling.

Since agricultural disturbance began late in the study area (ca. 1.6 ka BP), our pollen data demonstrated only climate-induced vegetation shifts during the entire period of Korean SPD data (5.9 ka to 2.2 ka BP). The co-occurrence of the decreases in AP values and SPD values implies that the change in the number of dwelling structures was attributable to late Holocene abrupt events. Although it is unknown whether the change was due to depopulation, elevated mobility, or both, remarkable drops in SPD values from ca. 2.8 ka to ca. 2.3 ka BP are more likely suggestive of a dramatic fall in population during this period.

From ca. 3 ka BP, in southwestern Korea, the Songguk-ri culture flourished based on rice agriculture. However, it seems to have completely fallen ca. 2.3 ka BP, as reflected in the SPD data. Korean archaeologists have not reached an agreement as to what caused such a mysterious decline of the prosperous Songguk-ri culture^103^, although Ahn and Hwang^104^ recently hypothesized that climate shifts may have been a possible culprit. Our AP records and Korean SPD data suggest that the disappearance of this culture may have been induced by climate deterioration from 2.8 ka to 2.3 ka BP.

Given the strong linkages between Yayoi culture in Japan and Songguk-ri culture, it would also be plausible to infer that Korean people migrated southward to find wetter areas suitable for growing rice, and even crossed the sea to Japan. The Yayoi period remains undefined as new radiocarbon results keep pushing its start backward^105^. Recently, Shoda^106^ made a reasonable argument based on radiocarbon dates from Korean sites that the Yayoi culture began ca. 2.8 ka BP with the arrival of people from southwestern Korea. Modern Japanese are largely descendants of the Yayoi people (more than 80% generic contribution)^107^. Thus, the possible role of an abrupt climate shift in this migration deserves further careful examination.

The 2.8 ka event and its impact on ancient societies have been actively explored in Europe^108,109^, but not in coastal East Asia, which is mostly due to the difficulty in finding undisturbed sample materials appropriate for paleoclimate studies. Our study for the first time sheds light on cultural responses to late Holocene cooling events other than the 4.2 ka event and the LIA in coastal East Asia.

## Conclusions

In this study, we investigated Holocene abrupt cooling events and the HCO based on a new high-resolution multi-proxy record from the south coast of Korea. A possible societal impact of the events is also explored using archaeological SPD data. The major findings from this study are summarized below.

1. Our pollen records (AP values) clearly demonstrate drying and/or cooling events centered at 9.8 ka, 9.2 ka, 8.2 ka, 4.7 ka, 4.2 ka, 3.7 ka, 3.2 ka, 2.8 ka, and 2.4 ka BP.
2. The AP data also indicate that forests were seriously damaged by a two-step successive drying during the period of 8.4 ka to 8 ka BP, and the HCO lasted from ca. 7.6 ka to ca. 4.8 ka BP.
3. Correlation analysis results indicated that during the past ~5500 years, climate shifts on the Korean peninsula were presumably driven by variations in WTP SST.
4. Simultaneous declines in the SPD and AP values from 2.8 ka to 2.3 ka BP may be indicative of a dramatic depopulation arising from abrupt climate deterioration. Refugee agriculturalists might have migrated southward from the peninsula and established the Yayoi culture in Japan. The 2.8 ka event and its cultural influence is recognized clearly for the first time in coastal East Asia.

## Materials and Methods

### Core materials and chronology

In 2017, a 32-m-long sediment core (STP17-013; hereafter GY-1) in 1-m-long sections was extracted from a reclaimed paddy field in the study area using a hydraulic corer (35°00′20.48″N, 127°46′40.94″E; Fig. 1). The uppermost section (0–1-m depth) and lowermost section (29–32-m depth) of the sediment core were not analyzed because the former appeared to have been disturbed by agricultural activities, and the latter consisted mostly of gravels (Fig. 2). Nine vegetation samples were submitted to the Korea Institute of Geosciences and Mineral Resources for accelerator mass spectrometry radiocarbon dating (Table 1). The calibrated age range was determined using the Clam 2.2 software^110^ and the IntCal13 dataset^111^.

### Multiproxy data

A total of 176 samples were taken for pollen analysis at intervals of 1–40 cm. Pollen was extracted using standard palynological procedures^94^. The samples were successively treated with HCl, KOH, HF, and acetolysis and sieved through 180- and 10-μm mesh filters to remove large organic debris and fine fractions after the KOH treatment. Pollen counts were obtained using a Leica microscope with a 40× objective lens at a total magnification of 400×. A minimum of 300 pollen grains were counted from each slide. However, five samples (at 100, 140, 180, 810, and 1060 cm) lacked sufficient pollen concentrations to permit counting to 300. In those samples, a minimum of 100, 150, or 200 pollen grains were counted.

A pollen diagram was produced using *Tilia*. *Lycopodium* spore tablets were added to each sample to calculate pollen concentrations. The total sum of the non-aquatic pollen and spores was used as the basis to calculate all percentages shown in the pollen diagram. A stratigraphically constrained cluster analysis was also conducted based on the percentages of non-aquatic taxa using CONISS, and four stratigraphic zones were delineated.

Mean grain size was measured at 10-cm intervals using a Mastersizer 2000 laser diffraction particle size analyzer (Malvern Instruments, Malvern, UK). Grain size values are represented in phi units (ϕ = −log_2_ (mean diameter in millimeters)). For this analysis, approximately 300 mg of sample was treated with 35% H_2_O_2_ to remove organic matter, and with 1 N HCl in a hot water bath to remove carbonates. For the elemental analyses, we took additional samples at the same depths used for the pollen analysis. These samples were treated with 5% HCl to remove carbonates prior to analysis. TOC and TN were measured using a Flash EA 2000 elemental analyzer (Thermo Scientific) at the National Center for Inter-University Research Facilities, Seoul National University, and the C/N ratio was calculated.

### Statistical analyses

An autospectral analysis on the arboreal pollen percentage (AP) was performed using the REDFIT program^112^. Three Welch-overlapped segment-averaging segments (N50 = 3), three degrees of freedom (dof = 3), 1000 Monte Carlo simulations (Nsim = 1000), and one Welch window (Iwin = 1) were applied for all analyses. The uppermost pollen zone exhibiting agricultural disturbance was excluded from the analysis. Thus, the AP records had 169 data points with a mean interval of ~50 years between points [t(1) = 1658 cal yr BP and t(169) = 10,020 cal yr BP]. A cross-spectral analysis was also performed between our AP data and Dongge Cave stalagmite δ^18^O data^57^ and between the AP data and the western tropical Pacific (WTP) SST reconstruction^58^ using the REDFIT-X software^113^ to assess the periodicities with significant coherency.

For correlation analyses, Dongge δ^18^O data and WTP SST data were resampled to the time points of the AP records (*n* = 169). Then, three proxy records were detrended using a singular spectrum analysis. Detrended values of Dongge δ^18^O, WTP SST, and our AP data were then smoothed using 10-point (~170 years), 5-point (~240 years), and 3-point (~150 years) moving averages, respectively. They were then normalized to the standard Z-scores for comparison. The 95% confidence intervals for the correlation coefficients among the three normalized proxy datasets were calculated using the Pearson T3 program, which was designed to estimate correlations between unevenly spaced autocorrelated data^114^.

## Data Availability

The datasets generated and/or analyzed during the current study are available from the corresponding author on reasonable request
